# Case Report: Precision COVID-19 Immunization Strategy to Overcome Individual Fragility: A Case of Generalized Lipodystrophy Type 4

**DOI:** 10.3389/fimmu.2022.869042

**Published:** 2022-04-06

**Authors:** Salvatore Zaffina, Eva Piano Mortari, Reparata Rosa Di Prinzio, Marco Cappa, Antonio Novelli, Emanuele Agolini, Massimiliano Raponi, Bruno Dallapiccola, Franco Locatelli, Carlo Federico Perno, Rita Carsetti

**Affiliations:** ^1^ Occupational Medicine/Health Technology Assessment and Safety Research Unit, Clinical-Technological Innovations Research Area, Bambino Gesù Children’s Hospital, IRCCS, Rome, Italy; ^2^ Diagnostic Immunology Research Unit, Multimodal Medicine Research Area, Bambino Gesù Children’s Hospital, IRCCS, Rome, Italy; ^3^ Unit of Endocrinology, Bambino Gesù Children’s Hospital, IRCCS, Rome, Italy; ^4^ Laboratory of Medical Genetics, Bambino Gesù Children’s Hospital, IRCCS, Rome, Italy; ^5^ Medical Direction, Bambino Gesù Children’s Hospital, IRCCS, Rome, Italy; ^6^ Genetics and Rare Diseases Research Division, Bambino Gesù Children’s Hospital, IRCCS, Rome, Italy; ^7^ Department of Pediatric Hematology and Oncology, Bambino Gesù Children’s Hospital, IRCCS, Rome, Italy; ^8^ Sapienza, University of Rome, Rome, Italy; ^9^ Microbiology and Diagnostic Immunology Unit and Multimodal Medicine Research Area, Bambino Gesù Children’s Hospital, IRCCS, Rome, Italy

**Keywords:** SARS-CoV-2 vaccine, *CAVIN1*, memory B cells, congenital generalized lipodystrophy type 4, case report

## Abstract

A 48-year-old patient affected with congenital generalized lipodystrophy type 4 failed to respond to two doses of the BNT162b2 vaccine, consisting of lipid nanoparticle encapsulated mRNA. As the disease is caused by biallelic variants of *CAVIN1*, a molecule indispensable for lipid endocytosis and regulation, we complemented the vaccination cycle with a single dose of the Ad26.COV2 vaccine. Adenovirus-based vaccine entry is mediated by the interaction with adenovirus receptors and transport occurs in clathrin-coated pits. Ten days after Ad26.COV2 administration, S- and RBD-specific antibodies and high-affinity memory B cells increased significantly to values close to those observed in Health Care Worker controls.

## Introduction

Congenital generalized lipodystrophy type 4 (CGL4) is an autosomal recessive disorder characterized by a massive loss of adipose tissue (1). The disease is caused by homozygous or compound heterozygous variants in the *CAVIN1* gene encoding for CAV1, a molecule indispensable for the biogenesis of caveolae (2). Caveolae are cell membrane invaginations with diverse functions, including endocytosis, lipid regulation, compartmentalization of signalling pathways and calcium signaling (3). Each caveola has an estimated number of 140–150 CAV1 molecules that cooperate with other members of the caveolin family (CAV2 and CAV3) (4). Deletion of CAV1 leads to a drastic reduction of all other caveolins and loss of caveolae. Patients with CAV1 mutations suffer from lipodystrophy, muscular dystrophy, cardiac arrhythmia, and osteoporosis, emphasizing the broad range of conditions associated with caveolae loss (5).

The COVID-19 pandemic has led to the accelerated development of anti-SARS-CoV-2 vaccines. The first vaccines approved for administration in humans are based on the use of mRNA or adenoviral vectors that force human cells to express the viral spike (S) protein and elicit both a B- and T-cell immune response (6, 7). mRNA vaccines are composed of codon-optimized sequences for the expression of the full-length S protein encapsulated in lipid nanoparticles (LNPs) (8). The LNPs have the function of protecting mRNA from degradation and facilitating its uptake, transport, and release inside the cell (9). In adenoviral vectors, the full-length SARS-CoV-2 S DNA replaces the early adenoviral genes E1 thus generating a replication-defective vector (9). Real-world studies have confirmed the effectiveness of all authorized mRNA and adenoviral vaccines against SARS-CoV-2 (10).

In our hospital a complete BNT162b2 mRNA vaccine cycle was administered to all Health Care Workers (HCWs) and employees, recently followed by a third booster dose. We previously showed that whereas serum specific antibodies decline with time, memory B cells (MBCs) continue to increase months after the last dose (11).

We report on a subject with CGL4 who failed to respond to a complete cycle with the BNT162b2 mRNA vaccine but mounted a normal response to a single dose of the Ad26.COV2.S vaccine.

## Case Presentation

A 48-year-old Caucasian male hospital employee came to our attention in May 2021 for failing to respond to the complete cycle of anti-SARS-CoV-2 BNT162b2 mRNA vaccine. The patient is affected by CGL4 resulting from a c.526G>T (p.Glu188Ter) homozygous variant in the *CAVIN1* gene. He has a complex clinical phenotype including extreme reduction of white fat, hypertrophic cardiomyopathy with recurrent atrial fibrillation and severe chronic respiratory failure. The multi-organ symptoms and complications reported in **Table 1** do not include increased susceptibility to infection, reduction of serum antibodies or impaired response to vaccination.

**Table 1:** Clinical history and response to vaccinations.

**Pathological remote history**	Cardiac problems: hypertrophic cardiomyopathy with recurrent atrial fibrillation since early age, undergone to multiple cardiac ablations; iron deficiency anaemia (treated with martial therapy)
	Respiratory problems: severe chronic respiratory failure
	Gastrointestinal problems: oesophageal achalasia with megaoesophagus (repeatedly treated with dilations and cardial infiltrations of botulinum toxin); hiatal hernia; hypomotile intestinal loops, constipation, recurrent gastrointestinal infections, haemorrhoidal prolapse, and proctitis; mild hepatic steatosis; liver abscess (treated with surgical drainage); recurrent cholestatic pancreatitis with secondary pancreatic insufficiency (treated with ERCP stenting)
	Muscle-skeletal problems: severe left-convex lumbar rotoscoliosis with dorsal compensation curve (angular value of 80° for the D12-L5 tract), lumbar lordosis and dorsal kyphosis; severe osteoporosis (BMD-DEXA: -3.7 SD for lumbar point and -4 SD for femur point); multiple vertebral collapses; muscle hypertrophy with bone cysts
	Ocular problems: keratoconus
	Carbohydrate intolerance due to a documented insulin resistance
**General therapy**	Replacement therapy with leptin (started a year ago), reduced to overcome the incoming carbohydrate intolerance
**Past vaccinations antibody titres**	• IgG against *Clostridium tetani*: 1.04 IU/mL (n.v. for sufficient immunity:0.6-1.0) • IgG against *Streptococcus pneumoniae*: 101 mg/L (n.v. for immunity: >35) • IgG against *Haemophilus influenzae*: >9 mg/L (n.v. for high immunity: >5.6)
**CD19+ B cells, % of lymphocytes, median (IQR 25^th^-75^th^)**	• 4.9 (4.6-6.9) • HCWs frequency: 8.03 (5.45-10.35)
**CD19+CD24+CD27+ memory B cells, % of B cells, median (IQR 25^th^-75^th^)**	• 44.45 (42.63-47.8) • HCWs frequency: 41.5 (31.7-47.9)

ERCP, Endoscopic Retrograde Cholangiopancreatography; BMD-DEXA, Bone Mineral Density- Dual-Energy X-ray Absorptiometry; n.v., normal value; SD, Standard Deviation.

**Figure 1:**
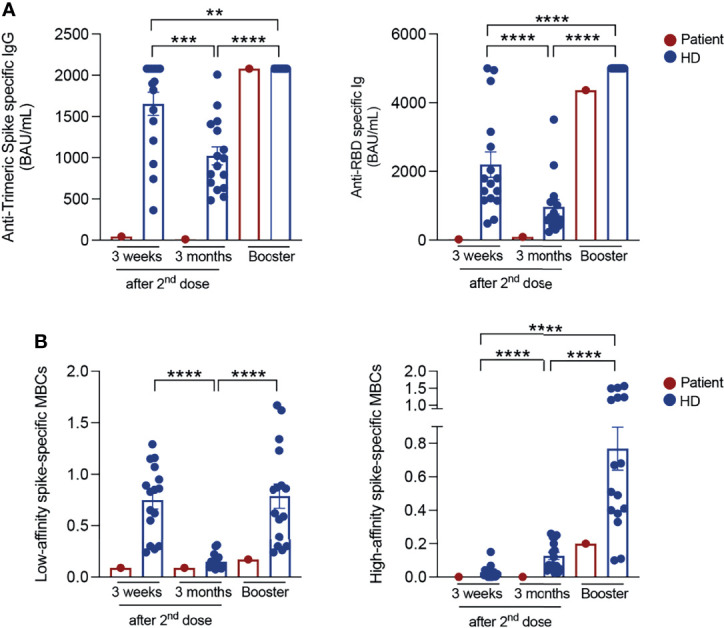
SARS-CoV-2 antibody responses and specific memory B cells. **(A)** Anti-Trimeric Spike specific IgG, total anti-RBD antibody levels and **(B)** percentage of S+ (low-affinity) and S++ (high-affinity) MBCs measured at different time points in vaccinated HCWs (in blue; *n=16*) and in the patient (in red): 3 weeks and 3 months after the second dose and 10 days post-booster (BNT162b2 mRNA vaccine for control HCWs and Ad26.COV2.S COVID-19 vaccine for the patient). Column indicate mean and SEM. Statistical significance was determined using Wilcoxon matched pairs signed rank test. **p < 0.01, ***p < 0.001, ****p < 0.0001.

Repeated serum antibody measurements showed a minimal response to the BNT162b2 mRNA vaccine as compared to healthy control HCWs (**Figure 1A**). Three weeks after the second dose, anti-Trimeric S IgG were 45.5 BAU/mL compared to a median value of 1911 BAU/mL (IQR: 1266-2080) in control HCWs. Three months after the last vaccine dose, titres decreased to 10.45 BAU/mL (control HCWs median: 947.7 (IQR: 648.1-1387)). Total anti-RBD antibodies were 23.1 BAU/ml three weeks after completion of vaccine cycle and 93.9 BAU/mL after three months, both values being much lower than those of the controls (control HCWs medians: 1717 BAU/mL (IQR: 1174-3044) at three weeks; 655 BAU/mL (IQR: 463.8-1156) at three months, **Figure 1A**). We previously demonstrated that it is possible to distinguish low-affinity (S+) and high-affinity (S++) Spike-specific MBCs by flow-cytometry (11). S++ MBCs are generated after the 2nd dose by a functional germinal center reaction (12, 13). In control HCWs, S+ MBCs were already detectable before vaccination and significantly expanded three weeks after completion of the immunization cycle (median 0.84% (IQR:0.36-1.04)). Three months after vaccination, the frequency of S+ MBCs decreased to 0.11% (IQR: 0.09-0.18) (**Figure 1B**).

High-affinity S++ MBCs that were found to be absent before vaccination, significantly increased 21 days after the second dose (control HCWs median: 0.016% (IQR:0.004-0.04)) and continued to significantly expand at later time point. Three months after the 2^nd^ dose, the median value of S++ MBCs was 0.09% (IQR 0.05-0.23) (11) (**Figure 1B**). In fully vaccinated HCWs, 20-25% of the S++MBCs was specific for the viral RBD (11).

In the peripheral blood of the patient, low-affinity S+ MBCs were detectable at all-time points. High-affinity S++ MBCs were below the limit of quantification and MBCs specific for the viral RBD were absent before and after vaccination.

LNPs can be internalized by different mechanisms, such as micropinocytosis, clathrin-mediated and caveolae-mediated endocytosis (14, 15). Known that CAV1 deficiency impairs the formation and function of caveolins (3), we hypothesized that the lack of caveolins may be responsible for the markedly reduced import of the vaccine LNPs, thus explaining the almost undetectable immune response of the patient. Therefore, according to the mix-and-match vaccination strategy, especially recommended for fragile patients (16), we decided to complement the vaccination cycle through the administration of a single dose of Ad26.COV2.S vaccine. After 10 days, antibody levels were comparable to those of all other HCWs (anti-Trimeric S: 2080 BAU/mL; anti-RBD: 4366 BAU/mL). High-affinity S++ MBCs also increased (0.2%) to a level similar to that observed in control HCWs.

## Discussion

It has been calculated that only in Europe nearly half a million lives have been saved by anti-SARS-CoV-2 vaccination in less than a year (17). This extraordinary success is due to the rapid development and availability of a new class of vaccines based on the administration of nucleic acids encoding the viral S protein. Both mRNA- and adenovirus-based vaccines instruct human cells to express the viral protein. Thus, human cells serve as immunogens and stimulate a potent and protective immune reaction (18, 19). Absent or insufficient response has been demonstrated in patients with alterations of the immune system due to treatment with immunosuppressive drugs, primary or secondary immunodeficiency and haematological malignancy (13, 20–23). Here we show lack of response to a complete vaccine cycle with the highly effective BNT162b2 mRNA vaccine in a subject with a CGL4 due to a homozygous pathogenic variant of *CAVIN1*. By abolishing caveolin formation and functions, CAV1 deficiency may severely impair the cellular import of the LNPs containing the immunizing mRNA. For the third dose the choice fell on the Ad26.COV2.S vaccine, based on the observation that adenovirus-based vectors do not requires caveolins for cellular entry and intracellular translocation (24, 25). The subsequent detection of a completely restored immune response is consistent with our hypothesis.

CGL4 is a rare multi-organ disease caused by defects of cell membrane-specialized structures that sense and import mediators of the intracellular environment destined to reach different intracellular vesicles compartment with multiple defined outcomes. Other rare diseases, included in the category of inborn error of metabolism, are due to mutations of molecules involved in intracellular vesicles function. Similar to the case described here, defect of vesicular structure or trafficking may impair intracellular import and transport of nucleic acid-based vaccines thus abolishing their function.

Patients with defects of caveolins or vesicular formation and transport may be unprotected notwithstanding the administration of the mRNA vaccines. Although antibody testing is not recommended to assess immunity after COVID-19 vaccination in the general population (26), our report shows that measurement of vaccine-induced specific antibodies may be an important tool to better care for fragile patients who may be not responder to immunization. Thanks to the different types of anti-COVID-19 vaccines already available or still in development, after exclusion of immunological deficiencies, it may be possible to identify the mechanisms leading to the failure to respond to a highly effective vaccine and a patient-centred counselling may be adopted to choose the best suitable immunization. Only patients unable to respond to all vaccine types will need to be treated with monoclonal antibodies to prevent COVID-19. COVID-19 clinical evolution has revealed rare genetic defects explaining disease resistance or extreme vulnerability (27, 28). Similarly, the study of the unexpected cases of vaccine failure may elucidate the intracellular mechanisms of vaccine function and guide the choice of the best immunization strategy for each patient thus overcoming individual fragilities. A wide range of vaccines acting with different mechanisms has been rapidly developed in response to the pandemic emergency. From this palette it will be possible to select the components of effective protocols for precision immunization and protection of patients with genetic diseases.

## Methods

### Vaccination

All HCWs and employees of the Bambino Gesù Children Hospital received two 30 μg doses of the BNT162b2 mRNA vaccine, 21 days apart, as prescribed by the immunization schedule. The efficacy of vaccination was evaluated in a monocentric observational study including 16 HCWs in good health conditions, randomly selected among the 3511 individuals working in our hospital. Serum and blood samples were collected at different time points before and after vaccination (11). HCWs and employees with known fragilities (primary or secondary immune deficiency, immunosuppressive treatment, haematological problems, or genetic diseases) were not included in the study but effectiveness of vaccination was measured and compared to control HCWs.

The Ethics Committee of Bambino Gesù Children Hospital, Rome, Italy, approved the studies on immune response (CE Nr: 291220) which were performed in accordance with the Good Clinical Practice guidelines, the International Conference on Harmonization guidelines, and the most recent version of the Declaration of Helsinki.

### Detection of Spike Specific Antibodies and Memory B Cells

Efficacy of vaccination was determined by the detection of antibodies against the receptor binding domain (RBD) of the virus, measurement of anti-Trimeric-S IgG in the serum and quantification of Spike-specific MBCs in the peripheral blood at different time points.

Semi-quantitative detection of total antibodies directed against the RBD of the virus S protein of SARS-CoV-2 was performed by an electro-chemiluminescence sandwich immunoassay (ECLIA), using Elecsys-anti SARS-CoV-2 S (Roche Diagnostics) test on a Cobas e801 analyser following the manufacturer’s instructions. Detection and quantification of anti-RBD antibodies were automatically calculated for each sample in U/mL, titres were deemed present if ≥ 0.8 U/mL. When antibody titre was > 250 U/mL, the instrument automatically diluted samples 20-fold, extending the upper limit for quantification to 5000 U/mL. U/mL were transformed to the Binding Arbitrary Unit (BAU)/mL of the first WHO International Standard for anti-SARS-CoV-2 immunoglobulins.

The quantitative determination of anti-Trimeric-S protein specific-IgG antibodies to SARS-CoV-2 was run on LiaisonXL platform by a new generation of chemiluminescence immunoassay (CLIA) TrimericS IgG assay (DiaSorin). The LIAISON^®^ SARS-CoV-2 TrimericS IgG assay measures between 4.81 and 2080 BAU/mL and titres were considered as present if ≥ 33.8 BAU/mL.

Detection of antigen-specific MBCs was performed as previously published (11–13, 20). Briefly, biotinylated recombinant SARS-CoV-2 Spike (S1+S2) was purchased from R&D and was individually multimerized with fluorescently-labelled streptavidin (PE and BUV395) at 4°C for one hour. Streptavidin PE-Cy7 (BD) was used as a decoy probe to gate out streptavidin-binding non-specific B-cells. ~4x10 (6) previously frozen PBMC samples were prepared and stained with antigen probe cocktail containing 100ng Spike per probe (total 200ng) and 2ng streptavidin-PE-Cy7 at 4°C for 30 min to ensure maximal staining quality. Following one wash step, surface staining was performed with labelled-antibodies (listed in table Antibody for staining) in Brilliant Buffer at 4°C for 30 min. Low-affinity and high-affinity Spike-specific MBCs were identified as CD19+CD24+CD27+CD38-Spike+ or Spike++ (**Supplementary Figure 1**). Samples were acquired on FACS LSRFortessa (BD) and analysed using FlowJo10.7.1 (BD). Limit of detection (LOD) and limit of quantification (LOQ) were calculated as previously reported (20, 29–31). Briefly, LOD was calculated as 20x100/total no. of events and LOQ was computed as 50x100/total no. of events (31).

Control HCWs were compared by the non-parametric Kruskal-Wallis test and with the Wilcoxon matched pair signed-rank test. Differences were deemed significant when P < 0.05. Statistical analyses were performed with GraphPad Prism 8.0 (GraphPad Software).

### Genetic Studies

After obtaining informed consent for the genetic analyses, clinical exome enrichment and parallel sequencing were performed on genomic DNA extracted from circulating leukocytes of the patient. Library preparation and clinical exome capture were performed by using the NimbleGen SeqCap Target Enrichment kit (Roche) according to the manufacture’s protocol and sequenced on the Illumina NextSeq 550 platform. The GenomeUp software (GenomeUp) was used for the variant calling and annotating variants. Sequencing data were aligned to the hg19 human reference genome. Functional impact of variants was analyzed by Combined Annotation Dependent Depletion (CADD) V.1.3, Sorting Intolerant from Tolerant (SIFT), and Polymorphism Phenotyping v2 (PolyPhen-2). Rare variants (MAF < 0.1%) were filtered based on the gnomAD database. Based on the guidelines of the American College of Medical Genetics and Genomics (S1), a minimum depth coverage of 30X was considered suitable for analysis. Variants were examined for coverage and Qscore (minimum threshold of 30), and visualized by the Integrative Genome Viewer (IGV). Clinical exome sequencing data analysis revealed a homozygous private nonsense change, NM_012232.5: c.526G>T (p.Glu188Ter) in *CAVIN1* gene, resulting in a premature stop codon.

## Data Availability Statement

The original contributions presented in the study are publicly available. This data can be found here: https://www.ebi.ac.uk/ena/browser/view/PRJEB50888.

## Ethics Statement

The Ethics Committee of Bambino Gesù Children Hospital, Rome, Italy, approved the studies on immune response (CE Nr: 291220). Written informed consent for participation was not required for this study in accordance with the national legislation and the institutional requirements.

## Author Contributions

SZ, EP, RP, and RC wrote the manuscript. EP performed experiments. EA and AN performed genetic studies. MC, FL, BD, and CP carefully corrected the manuscript. All authors contributed to the article and approved the submitted version.

## Funding

This work was funded by: Italian Ministry of Health RF2013-02358960 grant, Italian Ministry of Health COVID-2020-12371817 grant, grant “5 per mille, 2021” to RC.

## Conflict of Interest

The authors declare that the research was conducted in the absence of any commercial or financial relationships that could be construed as a potential conflict of interest.

## Publisher’s Note

All claims expressed in this article are solely those of the authors and do not necessarily represent those of their affiliated organizations, or those of the publisher, the editors and the reviewers. Any product that may be evaluated in this article, or claim that may be made by its manufacturer, is not guaranteed or endorsed by the publisher.

## Supplementary Material

Supplementary Figure 1 Gating strategy for the detection of memory B cells specific for the protein Spike. Samples were analysed three weeks and 3 months after second vaccine dose and after booster dose (BNT162b2 vaccine for control HCWs and Ad26.COV2.S vaccine for the patient). LOQ, Limit of Quantification.
