# Supplementary figures and images for: Case Report: Precision COVID-19 Immunization Strategy to Overcome Individual Fragility: A Case of Generalized Lipodystrophy Type 4
Source: Front Immunol. 2022 Apr 6;13:869042. doi: 10.3389/fimmu.2022.869042 (PMC9020769; doi:10.3389/fimmu.2022.869042)

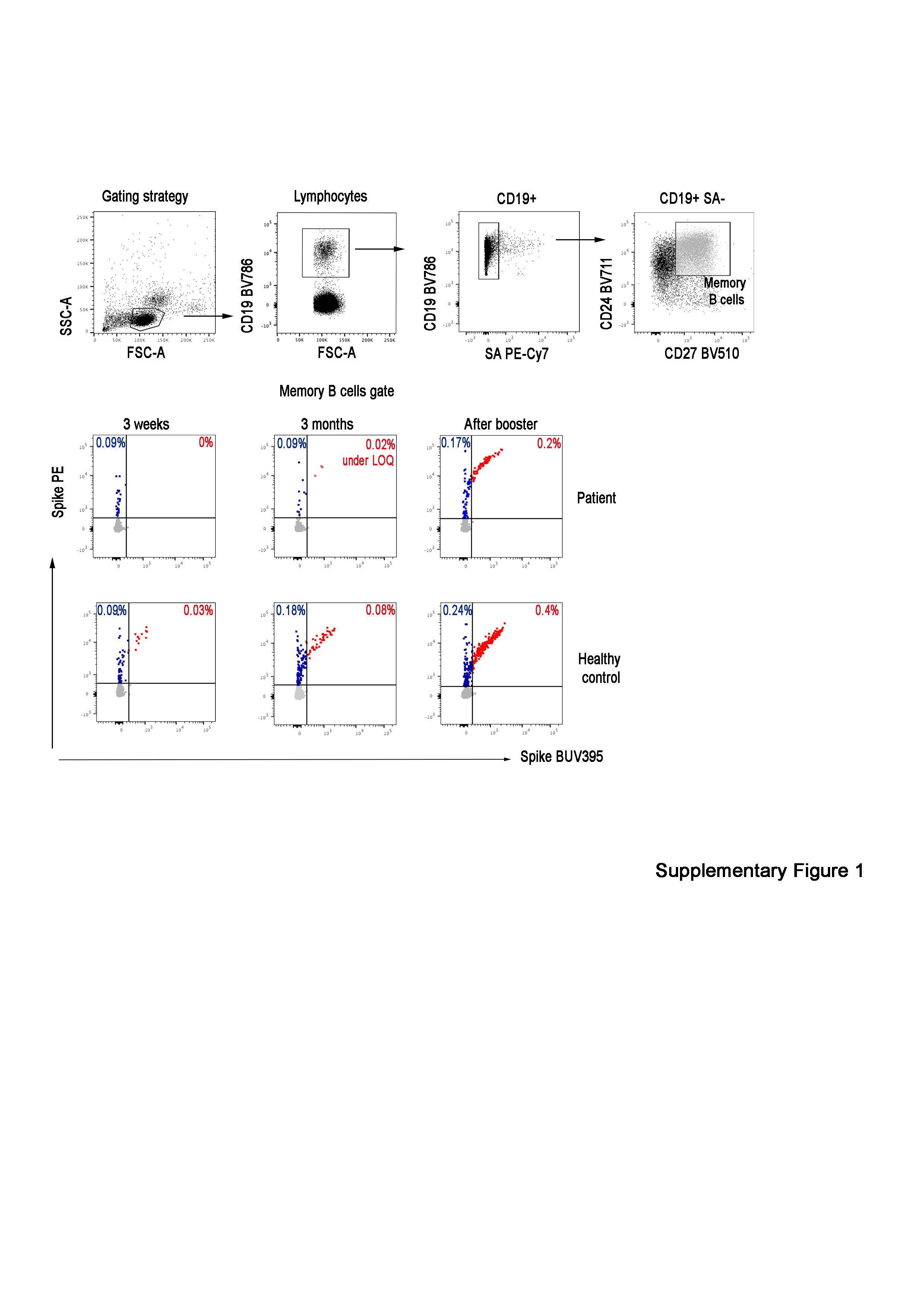

Supplement: Supplementary Figure 1 — Gating strategy for the detection of memory B cells specific for the protein Spike. Samples were analysed three weeks and 3 months after second vaccine dose and after booster dose (BNT162b2 vaccine for control HCWs and Ad26.COV2.S vaccine for the patient). LOQ, Limit of Quantification. [file Image_1.jpeg]
